# Seasonal Influenza Vaccine 2021/2022 Provides Limited Cross Reactivity Against Contemporary Swine Influenza A Virus Strains in Spain

**DOI:** 10.1111/irv.70158

**Published:** 2025-12-22

**Authors:** Paloma Encinas, Antonio Lalueza‐Blanco, Ana Isabel García‐Vaquero, Martha I. Nelson, Adolfo García‐Sastre, Gustavo del Real

**Affiliations:** ^1^ Department of Biotechnology National Institute of Agricultural and Food Research and Technology (INIA‐CSIC) Madrid Spain; ^2^ Facultad de Veterinaria Universidad Complutense de Madrid Madrid Spain; ^3^ Department of Internal Medicine Hospital Universitario 12 de Octubre Madrid Spain; ^4^ Occupational Health Department Hospital Universitario 12 de Octubre Madrid Spain; ^5^ Department of Microbiology Icahn School of Medicine at Mount Sinai New York New York USA; ^6^ National Center for Biotechnology Information National Library of Medicine National Institutes of Health Bethesda Maryland USA; ^7^ Global Health and Emerging Pathogens Institute Icahn School of Medicine at Mount Sinai New York New York USA; ^8^ Department of Medicine, Division of Infectious Diseases Icahn School of Medicine at Mount Sinai New York New York USA; ^9^ Tisch Cancer Institute Icahn School of Medicine at Mount Sinai New York New York USA; ^10^ Department of Pathology, Molecular and Cell‐Based Medicine Icahn School of Medicine at Mount Sinai New York New York USA; ^11^ Icahn Genomics Institute Icahn School of Medicine at Mount Sinai New York New York USA

**Keywords:** influenza A virus, pandemic risk, reverse zoonosis, swine, vaccine

## Abstract

Swine influenza A viruses (SIVs) pose a zoonotic risk, with variants detected in humans in Europe. This study evaluated the efficacy of the 2021/2022 seasonal influenza vaccine against SIVs. Forty‐six postvaccination human sera were tested via hemagglutination inhibition (HI) assay against Spanish SIV genotypes. Seroprotection rate (SPR, HI titer ≥ 40) was 76% (95% CI: 64–88) and 91% (95% CI: 83–99) for human vaccine strains H1 and H3, respectively. SPRs were 67% (95% CI: 53–81) for pandemic swine H1, 64% (95% CI: 50–78) for human seasonal‐like H1, 17%–46% for Eurasian avian‐like H1, 15% (95% CI: 5–25) for human seasonal‐like H3 from the 1970s, and 83%–93% for human seasonal‐like H3 from the 2000s SIVs.

## 1 Introduction

Influenza A virus (IAV) infects a wide range of hosts, including mammals and birds, causing seasonal or epidemic outbreaks. The hemagglutinin (HA), a surface glycoprotein of influenza viruses, mediates viral entry by binding to host cell receptors. It is the main antigen and the primary target of neutralizing antibodies [1]. Swine are a natural and highly relevant host for IAV, given that they have receptors for both human and avian influenza virus hemagglutinins. That is why pigs are considered the mixing vessel where avian and human‐origin influenza A strains interact and reassort, producing new viruses [2]. In fact, there have been numerous introductions of H1N1 and H3N2 IAVs into swine, which have given rise to a diverse set of IAV lineages: Eurasian avian‐like H1 (EAswH1, clade 1C), human seasonal‐like H1 (HUswH1, clade 1B), pandemic‐like H1 (PDMswH1, clade 1A), and human seasonal‐like H3 (HUswH3) from the 1970s or 2000s [3]. Swine IAV poses a threat to global public health due to their zoonotic potential to spill back into humans [2]. In fact, transmission of genetically diverse porcine IAV to humans has been reported worldwide, causing influenza variants [4], that in some cases may produce pandemic events, as occurred in the last influenza pandemic in 2009.

Influenza seasonal vaccines are updated annually for the north and south hemispheres according to the predicted most prevalent strains for the next season as recommended by the World Health Organization (WHO) [5]. Vaccines induce serum antibody production, mainly against viral HA, capable of inhibiting virus entry into host cells that limit the course of the infection [1]. Seasonal Influenza vaccine may provide some grade of cross protection to swine influenza A viruses (SIVs) [1]. This protection may not be enough in the case of a zoonotic influenza pandemic, so influenza candidate vaccine viruses (CVVs) are selected each year to match detected swine‐origin human Influenza variants [6]. One of the methodologies to contribute to determining the pandemic risk from swine‐origin IAVs is to assess the seroprotection rate (SPR) against current swine IAVs, using standardized methods like hemagglutination inhibition (HI) assay [7].

In this study, we aim to evaluate the SPR provided by the human seasonal influenza vaccine 2021/2022 against SIVs circulating in Spain. Furthermore, we examined the phylogenetic relationships between these SIVs and the swine‐origin influenza A variants detected in Europe since 2016, as well as their corresponding CVVs. This study provides relevant information for assessing the public health risk of zoonotic influenza caused by pigs and for the development of more effective broad‐spectrum CVVs.

## 2 Methods

### 2.1 Human Sera Samples

Postvaccination human sera were obtained from 46 healthy individuals who took part in the influenza seasonal vaccination campaign 2021/2022. Half men and half women, ages ranging from 25 to 69 years old (four individuals ≥ 65 years old) in Hospital 12 de Octubre in Madrid (Spain). Each participant received a single dose of inactivated influenza vaccine, which contained 15 μg of HA of each of the following influenza A strains: A/Victoria/2570/2019 (H1N1) pdm09, A/Cambodia/e0826360/2020 (H3N2), and B/Washington/02/2019 (B/Victoria). Serum samples from unvaccinated people (*n* = 6) were used as negative controls in the HI assay. Blood samples were collected by venipuncture 1 month after vaccination, and serum 0.4‐mL aliquots were frozen to −20° until use.

### 2.2 Hemagglutination‐Inhibition Assay

HI assay was performed according to standard procedures [8]. Briefly, serum samples were treated with receptor‐destroying enzyme (Sigma‐Merck, Germany) and adsorbed with packed turkey red blood cells. Sera were subjected to six serial twofold dilutions (1:20–1:640). HI assays were performed using “V” bottom microtiter plates (Nunc, Thermo Scientific). Results were considered positive if reciprocal serum dilution of 40 or above inhibited hemagglutination.

**FIGURE 1:**
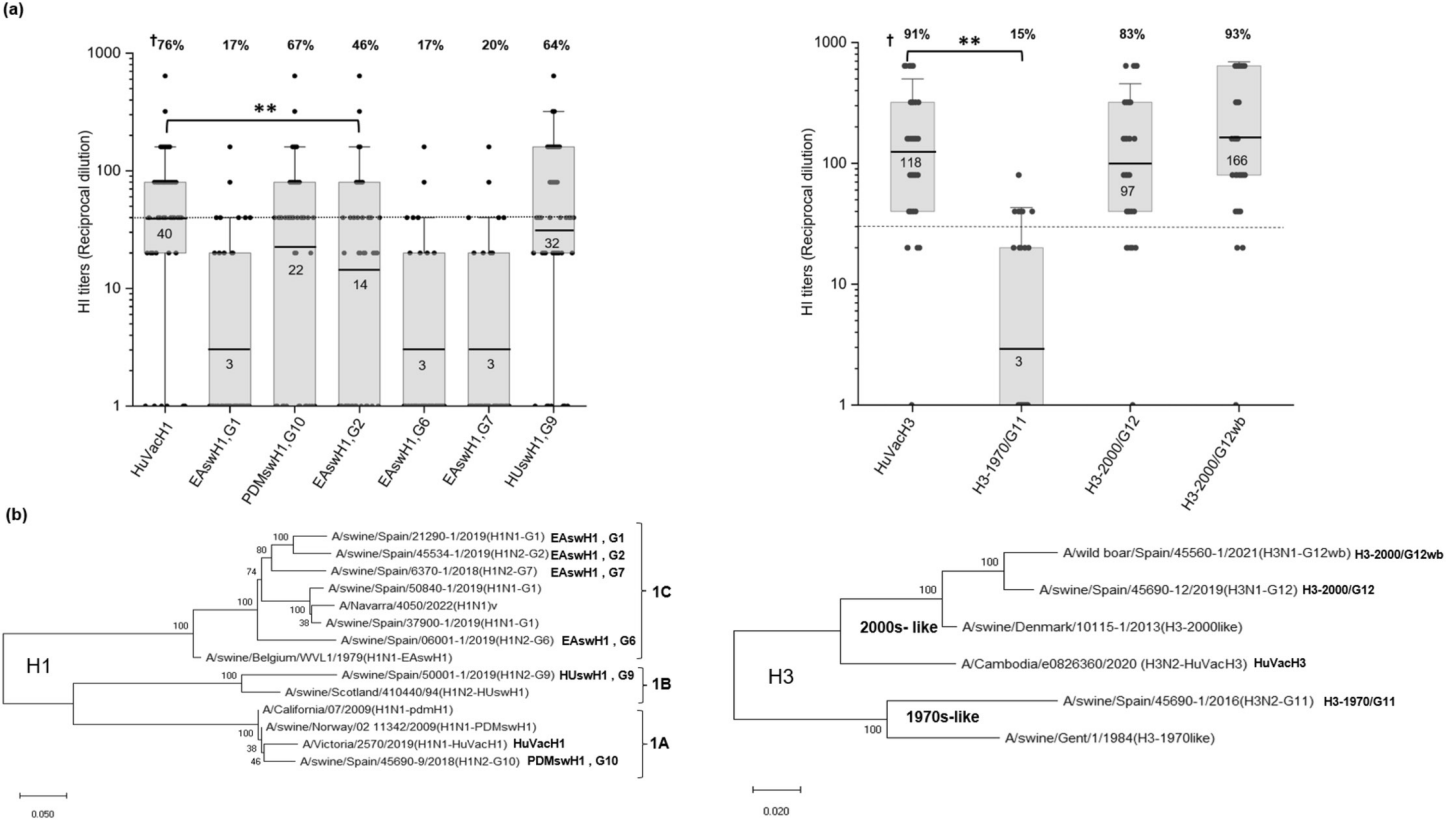
Hemagglutination inhibition (HI) assay results and phylogenetic relationship between strains. (a) Postvaccination serum HI titers of humans immunized with the seasonal influenza A vaccine 2021/2022 against contemporary Spanish swine influenza A viruses (SIVs). Boxes: interquartile range 25–75, vertical bars: standard deviation of the mean, line, and number inside the box: geometric mean of HI titer, dashed line: 1/40 serum dilution. ^†^Number at the top of the figure: percentage of vaccinated people with HI serum titer ≥ 1/40. **p < 0.01 Mann Whitney, G, indicates SIV genotype as previously described in Encinas et al. [9] (PMID: 35039784). (b) Maximum likelihood phylogenetic analysis of the amino acid sequences of the hemagglutinin (HA) of influenza A virus swine strains included in the study. H1 lineages: 1A, pandemic H1; 1B, human seasonal‐like H1; 1C, Eurasian avian‐like H1; H3 lineages: 1970s‐like, European human‐seasonal H3; 2000s‐like H3, novel human seasonal‐like H3, v: human influenza variant.

We previously identified genetically diverse SIV strains circulating in Spain (2015–2019) through routine surveillance. Influenza‐positive samples were isolated and propagated in cell culture and sequenced (Illumina). Twelve genotypes (G) were defined by lineage, clade, and phylogeny, including nine HA‐NA pairings, three internal cassettes, and four subtypes: H1N1, H1N2, H3N2, and H3N1 [9]. We selected nine representative genotypes with distinct HA‐NA combinations: A/swine/Spain/21290‐1/2019 (EAswH1, G1), 45534‐1/2019 (EAswH1, G2), 06001‐1/2019 (EAswH1, G6), 6370‐1/2018 (EAswH1, G7), 50001‐1/2019 (HUswH1, G9), 45690‐9/2018 (PDMswH1, G10), 45560‐1/2022 (HUswH3wb‐2000s, G12), 45690‐12/2019 (HUswH3‐2000s, G12), and 45690‐1/2016 (HUswH3‐1970s, G11). Two human vaccine strains (2021–2022)—A/Cambodia/e0826360/2020 (HuVacH3) and A/Victoria/2570/2019 (HuVacH1)—were used as positive controls. The phylogenetic relationship between strains, including representative viruses of the clade, is shown in Figure 1b, and strain details are found in Table S1. Geometric mean (GM), reciprocal HI values, and 95% confidence intervals (95% CI) were calculated using Origin2023b. Statistical differences in SPR between the swine and vaccine strains were assessed with the Mann–Whitney test (*p* < 0.05).

### 2.3 Phylogenetic and Genomic Sequence Analysis

Gene sequences of European human influenza variants (v) and WHO‐recommended zoonotic CVVs were retrieved from GISAID (Tables S2 and S3). Hemagglutinin phylogenies were inferred using the maximum likelihood (ML) method in MEGAX, applying a general time‐reversible (GTR) model with gamma‐distributed rate variation. Swine IAVs were included in the same analysis, and clades were assigned using the Swine Influenza Global Classification Tool (Bacterial and Viral Bioinformatics Resource Center). HA sequences were aligned with ClustalW; amino acid identity was calculated using BioEdit 7.7.1. Antigenic sites (Sa, Sb, Ca1, and Ca2) within the receptor binding site (RBS) were compared to identify amino acid substitutions. Antigenic site numbering is based on the complete HA sequence from the first methionine.

## 3 Results

### 3.1 Seroprotection Rates of Individuals Immunized With the Seasonal Influenza Vaccine 2021/2022 Against Swine Isolates

SPR, defined as the reciprocal dilution HI titer ≥ 40, was 76% (95% CI: 64–88) against HuVacH1 (GM: 40 [25–66]) and 91% (95% CI: 83–99) against HuVacH3 (GM: 118 [82–170]). Cross‐reactive antibodies were observed in 67% (95% CI: 53–81) against PDMswH1 (G10, GM: 22 [12–39]), and 64% (95% CI: 50–78) against HUswH1 (G9, GM: 32 [19–54]). Limited cross‐reactivity was found for EAswH1 strains: 17% (95% CI: 6–28) to G1 and G6, 20% (95% CI: 8–32) to G7 (GM: 3 [2–5]), and 46% (95% CI: 32–60) to G2 (GM: 14 [7–25]). Regarding H3 lineages, 83%–93% of individuals had cross‐reactive antibodies to novel 2000s‐like H3 strains (G12) (GM > 97), whereas only 15% (95% CI: 5–25) responded to the European 1970s‐like H3 strain (G11, GM: 3 [2–4]), significantly lower than the response to HuVacH3 (91%, GM: 118 [82–170]) (Figure 1a). Serum samples from unvaccinated individuals did not show HI antibodies to any of the viruses tested.

### 3.2 Phylogenetic and Genomic Sequence Analysis

**FIGURE 2:**
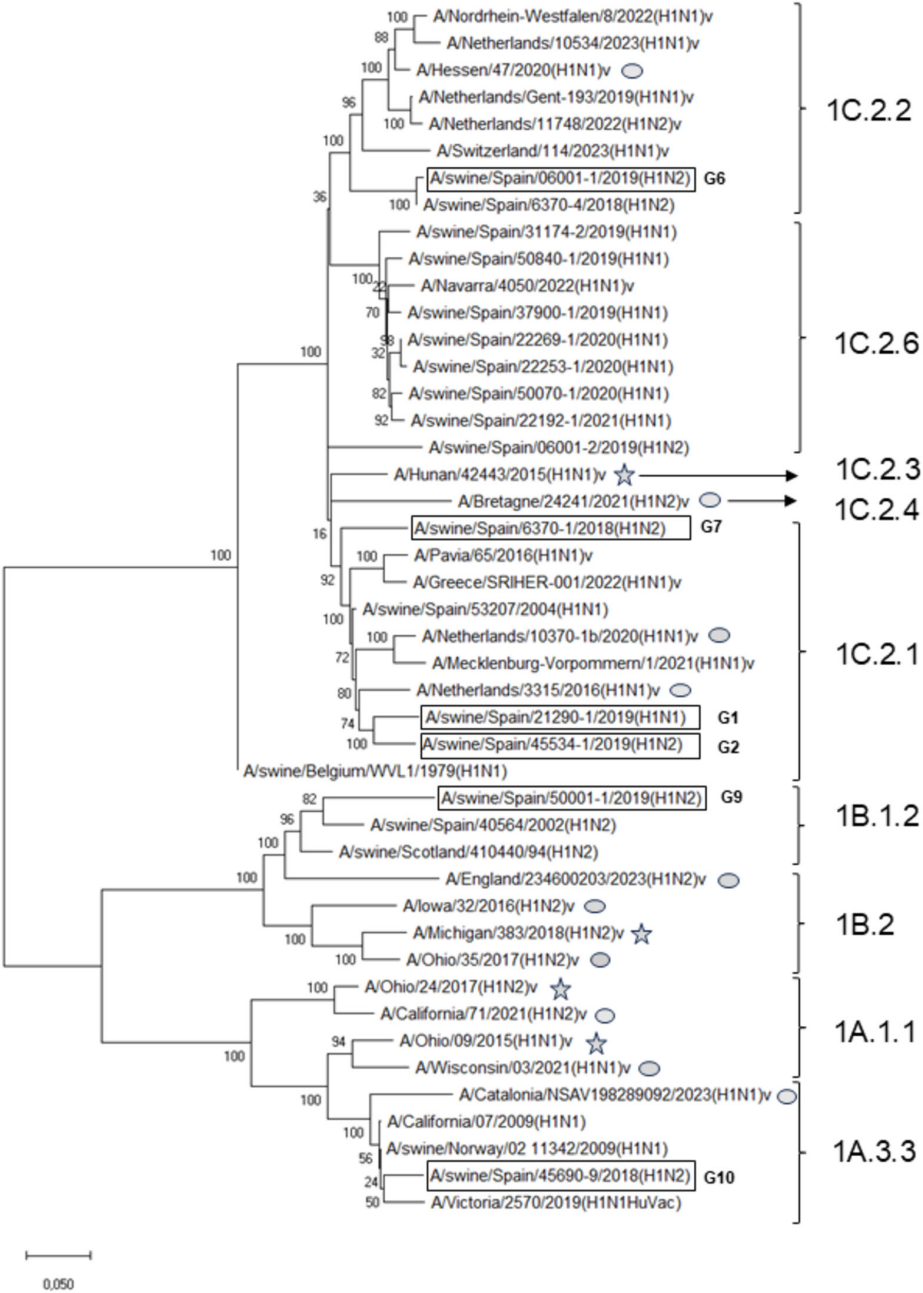
Phylogenetic analysis of influenza A viruses. Maximum likelihood phylogenetic tree of hemagglutinin 1 (HA1) nucleotide sequences from influenza A viruses included in this study. Star: prepared candidate vaccine viruses (CVVs); gray circles: in preparation CVVs; v: human variants detected in Europe since 2016; HuVac: human vaccine strain (H1), squared: swine influenza A viruses (SIVs) used in serological assays, G indicates SIV genotype as previously described in Encinas et al., 2022 (PMID: 35039784) [9].

Most human influenza A variants of swine origin detected in Europe since 2016 belong to the EAswH1 lineage, particularly clades 1C.2.1, 1C.2.2, and 1C.2.4 (Table S2 and Figure 2), with the exception of an H1N1v case (A/Navarra/4050/2022) obtained from a hospitalized patient in Navarra, Spain. The patient's serum was serotyped in our laboratory using two closely related Spanish SIVs from clade 1C.2.6: A/swine/Spain/37900‐1/2019 and A/swine/Spain/50840‐1/2019 (G1, Figure 1b), yielding HI titers of 360. Only two other recent human cases involved different lineages: H1N2v (HUswH1, 1B.1.1) in England and H1N1v (PDMswH1, 1A.3.3.2) in Spain. Most existing or in preparation CVVs target clades 1A and 1B, the dominant lineages in the United States, with some strains representing the EAswH1 lineage (Table S3 and Figure 2). No human H3N2 variants have been reported in Europe since 2016.

At the protein level, PDMswH1 shares 93% amino acid identity in the HA with HuVacH1, showing six substitutions in antigenic motifs, including three nonconservative changes (P154S, K173N, and Q180K) within the RBS (Table S4). Likewise, HuVacH3 is ~88% identical to 2000s‐like H3 G12 SIV (Y175F and K205N) but only 81% to 1970s‐like H3 G11 strains, which exhibit seven amino acid replacements in antigenic sites (Table S5). Regarding the four EAswH1 strains tested, several amino acid differences in key antigenic sites were observed: Sa (N142D, Q180K), Ca2 (P154S, K159N), Sb (K173N, A212N), and Ca1 (I183T) with amino acid identity below 77% (Table S4).

## 4 Discussion

Previous human‐to‐swine influenza spillovers [10] and the 2009 influenza pandemic highlight the importance of assessing the pandemic potential of emerging SIVs, considering both viral phenotypes and host immunity. Gaps in serologic immunity may increase the pandemic risk, as cross‐protective anti‐IAV antibodies are a key barrier to zoonotic transmission [11]. In this study, human antisera from individuals vaccinated during the 2021/2022 influenza season showed limited cross‐reactivity to Eurasian‐lineage SIVs.

The high SPR (83%–93%) to the novel human seasonal‐like H3 from the 2000s (G12) likely reflects similarity to the HuVacH3 hemagglutinin. This segment originated from humans, entering European swine in the 2000s [3] and undergoing minimal genetic drift, although two amino acid substitutions (Y175F and K205N) were observed in antigenic motifs. This high SPR may explain the absence of human H3 variants in Europe since 2016, in contrast to the United States [12].

The second‐highest SPR (67%) was observed against the PDMswH1 strain, which shares most RBS amino acids with HuVacH1, except for P154S, K173N, and Q180K, nonconservative substitutions that may impair antibody recognition and reduce HI titer. A 64% SPR was observed against HUswH1 (G9), an enzootic SIV introduced into European pigs in the 1990s [3]. This may reflect immune memory, as all study participants were > 22 years old. Supporting this, a prior study found higher seroprevalence (62%) to H1 SIV of lineage 1B.1.2.1 in individuals born before 1996 versus 1997–2017 [13].

The most common H1 influenza human variant of swine origin detected in Europe since 2016 belongs to the Eurasian avian‐like H1 lineage. In 2022, a human infection in Spain was linked to clade 1C.2.6 SIV, consistent with the dominance of this lineage in European pigs [14]. However, the seasonal influenza A vaccine elicited limited cross‐reactive antibodies to several genotypes of this lineage. These findings agree with previous data showing that ferrets vaccinated with the 2016–2017 seasonal vaccine were protected against clade 1A pandemic H1 but not against clade 1C SIVs [15]. A single RBS mutation can reduce HI titers by ≥ 8‐fold using monoclonal antibodies [16]. Sequence analysis of the four EAswH1 strains tested revealed amino acid differences in key antigenic sites: Sa (N142D, Q180K), Ca2 (P154S, K159N), Sb (K173N, A212N), and Ca1 (I183T), which may explain SPR variation in human sera.

We did not assess antibodies targeting the HA stalk or other IAV antigens such as neuraminidase (NA), which may also contribute to serological immunity against infection [1, 7]. Nonetheless, HI assays are used by the WHO as a measure of serological immunity in risk assessment tools for IAVs as a correlate of protection against influenza infection [7]. This study could have benefited from the inclusion of CVVs in the HI assays; however, this was not feasible due to availability constraints. The serum samples here analyzed reflect a cohort of vaccinated adult volunteers from the 2021/2022 seasonal influenza campaign in Spain.

In this study, we show that influenza vaccinated patients showed minimal to no antibody cross‐reactivity with SIVs from the Eurasian avian‐like H1 lineage, the most prevalent human variant influenza strain detected in Europe since 2016, and the European 1970s‐like H3 lineage. This gap in serologic immunity could increase the probability of a pandemic if SIVs from these lineages become transmissible in humans. Therefore, human influenza pandemic plans must take into account this possibility.

## Author Contributions

**Paloma Encinas:** investigation, methodology, software, data curation, supervision, formal analysis, validation, writing – original draft, writing – review and editing. **Antonio Lalueza‐Blanco:** methodology, data curation. **Ana Isabel García‐Vaquero:** methodology, data curation. **Martha I. Nelson:** methodology, supervision, validation, visualization, writing – review and editing. **Adolfo García‐Sastre:** supervision, investigation, funding acquisition, visualization, project administration, resources, writing – review and editing, validation. **Gustavo del Real:** conceptualization, methodology, data curation, supervision, formal analysis, validation, investigation, funding acquisition, project administration, visualization, resources, writing – original draft, writing – review and editing.

## Ethics Statement

Official ethical approval was approved prior to the procedures by the Ethical Committee of the Hospital 12 de Octubre (No. CEIm: 22/218).

## Consent

All participants were informed of the procedure and purpose of the study, and a written consent was signed. The usage of the samples was offered in three levels (project, collection, or biobank) so each participant could choose their own option. Sera samples were anonymized to preserve the intimacy of participants.

## Conflicts of Interest

The A.G.‐S. laboratory has received research support from GSK, Pfizer, Senhwa Biosciences, Kenall Manufacturing, Blade Therapeutics, Avimex, Johnson & Johnson, Dynavax, 7Hills Pharma, Pharmamar, ImmunityBio, Accurius, Nanocomposix, Hexamer, N‐fold LLC, Model Medicines, Atea Pharma, Applied Biological Laboratories, and Merck. A.G.‐S. has consulting agreements for the following companies involving cash and/or stock: Castlevax, Amovir, Vivaldi Biosciences, Contrafect, 7Hills Pharma, Avimex, Pagoda, Accurius, Esperovax, Applied Biological Laboratories, Pharmamar, CureLab Oncology, CureLab Veterinary, Synairgen, Paratus, Pfizer, Virofend, and Prosetta. A.G.‐S. has been an invited speaker in meeting events organized by Seqirus, Janssen, Abbott, Astrazeneca, and Novavax. A.G.‐S. is an inventor on patents and patent applications on the use of antivirals and vaccines for the treatment and prevention of virus infections and cancer, owned by the Icahn School of Medicine at Mount Sinai, New York. All other authors declare no conflicts of interest.

## Supporting information


**Table S1:** List of influenza A strains used in this work. ^†^ Inactivated human seasonal vaccine strains 2021/2022, ^‡^contemporary swine H1 influenza A strains, ^§^contemporary swine H3 influenza A strains, Influenza A virus genotypes previously described in Encinas et al., 2022, PMID: 35039784.
**Table S2:** Influenza A variants of swine origin detected in Europe since 2016. ^†^Candidate vaccine strains in preparation.
**Table S3:** Availability of candidate vaccine viruses (CVVs) against influenza A (H1) at 23th February 2024. v: human Influenza variants
**Table S4:** Hemagglutinin (HA) 1 antigenic variation. Amino acid substitutions in HA1 between the human seasonal vaccine strain pdmH1N1 (HuVacH1), contemporary swine influenza strains, and a Spanish influenza variant are shown. G: influenza A virus genotypes previously described in Encinas et al., 2022, PMID: 35039784. Percent identity refers to the full‐length HA protein. Substitutions at antigenic sites (Sa, Ca1, Ca2, Sb) within the receptor binding domain (RBD) are indicated. ^†^Antigenic site numbering is based on the complete HA sequence from the first methionine. Conservative substitutions are shaded dark grey; non‐conservative substitutions are shaded light grey. White boxes correspond to identical residues.
**Table S5:** Hemagglutinin (HA) 3 antigenic variation. Amino acid substitutions in HA3 of contemporary swine influenza strains are shown in comparison to the human seasonal H3N2 vaccine strain (HuVacH3). G: influenza A virus genotypes previously described in Encinas et al., 2022, PMID: 35039784. Percent identity refers to the full‐length HA protein. Substitutions are indicated at antigenic sites within the receptor binding domain (RBD), specifically at H3‐numbered positions: 145, 155, 156, 158, 159, 189, and 193. ^†^ Antigenic site numbering based on the complete HA sequence from the first methionine. Conservative substitutions are shaded dark grey; non‐conservative substitutions are shaded light grey. White boxes correspond to identical residues.

## Data Availability

The data that support the findings of this study are available from the corresponding author upon reasonable request.
