# Supplementary material for: Seasonal Influenza Vaccine 2021/2022 Provides Limited Cross Reactivity Against Contemporary Swine Influenza A Virus Strains in Spain
Source: Influenza Other Respir Viruses. 2025 Dec 22;19(12):e70158. doi: 10.1111/irv.70158 (PMC12981525; doi:10.1111/irv.70158)
Supplement: Supplementary file 1 — Table S1: List of influenza A strains used in this work. † Inactivated human seasonal vaccine strains 2021/2022, ‡contemporary swine H1 influenza A strains, §contemporary swine H3 influenza A strains, Influenza A virus genotypes previously described in Encinas et al., 2022, PMID: 35039784. Table S2: Influenza A variants of swine origin detected in Europe since 2016. †Candidate vaccine strains in preparation. Table S3: Availability of candidate vaccine viruses (CVVs) against influenza A (H1) at 23th February 2024. v: human Influenza variants Table S4: Hemagglutinin (HA) 1 antigenic variation. Amino acid substitutions in HA1 between the human seasonal vaccine strain pdmH1N1 (HuVacH1), contemporary swine influenza strains, and a Spanish influenza variant are shown. G: influenza A virus genotypes previously described in Encinas et al., 2022, PMID: 35039784. Percent identity refers to the full‐length HA protein. Substitutions at antigenic sites (Sa, Ca1, Ca2, Sb) within the receptor binding domain (RBD) are indicated. †Antigenic site numbering is based on the complete HA sequence from the first methionine. Conservative substitutions are shaded dark grey; non‐conservative substitutions are shaded light grey. White boxes correspond to identical residues. Table S5: Hemagglutinin (HA) 3 antigenic variation. Amino acid substitutions in HA3 of contemporary swine influenza strains are shown in comparison to the human seasonal H3N2 vaccine strain (HuVacH3). G: influenza A virus genotypes previously described in Encinas et al., 2022, PMID: 35039784. Percent identity refers to the full‐length HA protein. Substitutions are indicated at antigenic sites within the receptor binding domain (RBD), specifically at H3‐numbered positions: 145, 155, 156, 158, 159, 189, and 193. † Antigenic site numbering based on the complete HA sequence from the first methionine. Conservative substitutions are shaded dark grey; non‐conservative substitutions are shaded light grey. White boxes correspond to [file IRV-19-e70158-s001.docx]

**SUPPLEMENTARY FILES**

**Table S1. List of influenza A strains used in this work. ^†^ Inactivated human seasonal vaccine strains 2021/2022, ^‡^contemporary swine H1 influenza A strains, ^§^contemporary swine H3 influenza A strains,** **Influenza A virus genotypes previously described in Encinas et al., 2022, PMID: 35039784.**

| **Ref** | **Name** | **Subtype** | **Lineage** | **Clade** | **Accession number** | **Genotype^¶^** |
| --- | --- | --- | --- | --- | --- | --- |
| **HuVac_H1^†^** | **A/Victoria/2570/2019** | **H1N1** | **H1pdm** | **1A.3.3.2** | **OQ719015.1** |  |
| **HuVac_H3^†^** | **A/Cambodia/e0826360/2020** | **H3N2** | **H3_2020** |  | **EPI1848532** |  |
| **45690-9/2018^‡^** | **A/swine/Spain/45690-9/2018** | **H1N2** | **H1pdm** | **1A.3.3.2** | **MZ945846.1** | **G10** |
| **6370-1/2018^‡^** | **A/swine/Spain/6370-1/2018** | **H1N2** | **EAswH1** | **1C.2.1** | **PQ047764.1** | **G7** |
| **21290-1/2019^‡^** | **A/swine/Spain/21290/2019** | **H1N1** | **EAswH1** | **1C.2.1** | **MZ373100.1** | **G1** |
| **06001-1/2019^‡^** | **A/swine/Spain/06001-1/2019** | **H1N2** | **EAswH1** | **1C.2.2** | **MZ373107.1** | **G6** |
| **45534-1/2019^‡^** | **A/swine/Spain/45534-1/2019** | **H1N2** | **EAswH1** | **1C.2.1** | **MZ945771.1** | **G2** |
| **50001-1/2019^‡^** | **A/swine/Spain/50001-1/2019** | **H1N2** | **HUswH1** | **1.B.1.2** | **MZ945794.1** | **G9** |
| **45560-1/2021^§^** | **A/wild boar/Spain/45560-1/2021** | **H3N1** | **H3_2000.3** |  | **PP338808.1** | **G12** |
| **45690-12/2019^§^** | **A/swine/Spain/45690-12/2019** | **H3N1** | **H3_2000.3** |  | **MZ363769.1** | **G12** |
| **45690-1/2016^§^** | **A/swine/Spain/45690-1/2016** | **H3N2** | **H3_1970.1** |  | **MF872855.1** | **G11** |

| **Strain name** | **Subtype** | **H1 Clade** | **Collection date** | **Country** | **Gender** | **Age(years)** | **HA_ID** |
| --- | --- | --- | --- | --- | --- | --- | --- |
| A/Pavia/65/2016 | H1N1v | 1C.2.1 | 01/10/2016 | Italy | Male | 40 | EPI1804905 |
| A/Netherlands/3315/2016^†^ | H1N1v | 1C.2.1 | 01/11/2016 | Netherlands | Female | 9 | EPI1804909 |
| A/Netherlands/Gent-193/2019 | H1N1v | 1C.2.2 | 24/09/2019 | Netherlands | Male | 43 | EPI1735009 |
| A/Hessen/47/2020^†^ | H1N1v | 1C.2.2 | 10/06/2020 | Germany | Male | 2 | EPI1757439 |
| A/Netherlands/10370-2/2020^†^ | H1N1v | 1C.2.1 | 24/09/2020 | Netherlands | Male | 31 | EPI1838453 |
| A/Mecklenburg-Vorpommern/1/2021 | H1N1v | 1C.2.1 | 19/04/2021 | Germany | Male | 17 | EPI1868537 |
| A/Bretagne/24241/2021^†^ | H1N2v | 1C.2.4 | 18/08/2021 | France | Male | - | EPI1913513 |
| A/Austria/1445532 | H1N2v | 1C.2.4 | 01/09/2021 | Austria | Male | - | EPI1937574 |
| A/Nordrhein-Westfalen/8/2022 | H1N1v | 1C.2.2 | 24/03/2022 | Germany | Male | 34 | EPI2030866 |
| A/Greece/SRIHER-001/2022 | H1N1v | 1C.2.1 | 26/06/2022 | Greece | Unknown | Unknown | EPI2645139 |
| A/Netherlands/11748/2022 | H1N2v | 1C.2.2 | 01/10/2022 | Netherlands | Female | 26 | EPI2193003 |
| A/Navarra/4050/2022 | H1N1v | 1C.2.6 | 17/10/2022 | Spain | Male | 63 | EPI2240803 |
| A/Netherlands/10534/2023 | H1N1v | 1C.2.2 | 21/08/2023 | Netherlands | Male | Not provided | EPI2721674 |
| A/England/234600203/2023^†^ | H1N2v | 1B.1.1 | 27/11/2023 | United Kingdom | Male | Not provided | EPI2805024 |
| A/Switzerland/114/2023 | H1N1v | 1C.2.2 | 27/11/2023 | Switzerland | Unknown | Unknown | EPI2816125 |
| A/Catalonia/NSAV198289092/2023^†^ | H1N1v | 1A.3.3.2 | 14/12/2023 | Spain | Unknown | Unknown | EPI2923381 |

**Table S2: Influenza A variants of swine origin detected in Europe since 2016. ^†^**Candidate vaccine strains in preparation.

**Table S3. Availability of candidate vaccine viruses (CVVs) against influenza A (H1) at 23th February 2024.** v: human Influenza variants

|  |  |  |  |
| --- | --- | --- | --- |
| **Already prepared** | **Isolate ID** | **HA GISAID** | **Lineage** |
| A/Ohio/09/2015 (H1N1)v | EPI_ISL_179404 | EPI590132 | 1A.3.3.3 |
| CNIC-1601(A/Hunan/42443/2015 ) (H1N1)v | EPI_ISL_539892 | EPI1804911 | 1C.2.3 |
| A/Michigan/383/2018 (H1N2)v | EPI_ISL_320690 | EPI1271066 | 1B.2.1 |
| A/Ohio/24/2017 (H1N2)v | EPI_ISL_277243 | EPI1056725 | 1A.1.1 |
| **In preparation** | **Isolate ID** | **HA GISAID** | **Lineage** |
| A/Catalonia/NSAV198289092/2023 (H1N1)v | EPI_ISL_18782577 | EPI2923381 | 1A.3.3.2 |
| A/England/234600203/2023 (H1N2)v | EPI_ISL_18548251 | EPI2805024 | 1B.1.1 |
| A/California/71/2021 (H1N2)v | EPI_ISL_10864794 | EPI1994277 | 1A.1.1.3 |
| A/Bretagne/24241/2021 (H1N2)v | EPI_ISL_4106156 | EPI1913513 | 1C.2.4 |
| A/Wisconsin/03/2021 (H1N1)v | EPI_ISL_2479994 | EPI1868840 | 1A.3.3.3 |
| A/Ohio/35/2017 (H1N2)v | EPI_ISL_277244 | EPI1056733 | 1B.2.1 |
| A/Iowa/32/2016 (H1N2)v | EPI_ISL_238918 | EPI864027 | 1B.2.2.1 |
| A/Netherlands/3315/2016 (H1N1)v | EPI_ISL_243093 | EPI888684 | 1C.2.1 |
| A/Hessen/47/2020 (H1N1)v | EPI_ISL_491173 | EPI1757439 | 1C.2.2 |
| A/Netherlands/10370-1b/2020 (H1N1)v | EPI_ISL_717720 | EPI1838437 | 1C.2.1 |

**Table S4.** **Hemagglutinin (HA) 1 antigenic variation**. Amino acid substitutions in HA1 between the human seasonal vaccine strain pdmH1N1 (HuVacH1), contemporary swine influenza strains, and a Spanish influenza variant are shown. G: influenza A virus genotypes previously described in Encinas et al., 2022, PMID: 35039784. Percent identity refers to the full-length HA protein. Substitutions at antigenic sites (Sa, Ca1, Ca2, Sb) within the receptor binding domain (RBD) are indicated. **^†^**Antigenic site numbering is based on the complete HA sequence from the first methionine. Conservative substitutions are shaded dark grey; non-conservative substitutions are shaded light grey. White boxes correspond to identical residues.

|  |  |  | **Antigenic motif** | | | | | | | | | | | | |
| --- | --- | --- | --- | --- | --- | --- | --- | --- | --- | --- | --- | --- | --- | --- | --- |
|  |  |  | **^†^Sa** | **Ca2** | **Ca2** | **Sa** | **Sb** | **Sa** | **Sa** | **Ca1** | **Ca1** | **Ca1** | **Sb** | **Sb** | **Ca2** |
| **Strain name** | **Clade** | **%Identity** | 142 | 154 | 159 | 172 | 173 | 179 | 180 | 183 | 187 | 196 | 207 | 212 | 239 |
| A/Victoria/2570/2019 (HuVacH1) | 1A.3.3.2 |  | N | P | K | G | K | N | Q | I | G | I | S | A | D |
| A/sw/Spain/45690-9/2018 (pdmH1N1, G10) | 1A.3.3.2 | 93% |  | S |  |  | N | S | K | V |  |  |  | V |  |
| A/sw/Spain/50001-1/2019 (H1N2, G9) | 1.B.1.2 | 77% | K | S | S | N | G | S | K | M | K |  | A | E |  |
| A/swine/Spain/6370-1/2018(H1N2, G7) | 1C.2.1 | 77% |  | S | N |  | N | S | N | T |  | V | T | N | E |
| A/swine/Spain/21290-1/2019(H1N1, G1) | 1C.2.1 | 76% | D | S | N |  | N | S | K | T |  | V | T | N | E |
| A/swine/Spain/06001-1/2019(H1N2, G6) | 1C.2.2 | 76% | D | S | N | N | N | R | K | T |  | V | T | N | E |
| A/swine/Spain/45534-1/2019(H1N2, G2) | 1C.2.1 | 77% |  | S | N |  | N | S | K | T |  | V | T | N | E |
| A/Navarra/4050/2022(H1 variant) | 1C.2.6 | 77% |  |  | N |  | N | S | K | T |  | V | T | N | E |

|  |  |  | **Antigenic motif** | | | | | | |
| --- | --- | --- | --- | --- | --- | --- | --- | --- | --- |
|  |  |  | **145** | **155** | **156** | **158** | **159** | **189** | **193** |
| **Strain name** | **Clade** | **%Identity** | **^†^**161 | 171 | 172 | 174 | 175 | 205 | 209 |
| **A/Cambodia/e0826360/2020 (HuVacH3)** | H3_2020 |  | S | T | H | N | Y | K | S |
| A/swine/Spain/45690-1/2016 (H3N2, G11) | H3_1970.1 | 81% | N | Y | K | G | N | R | N |
| A/swine/Spain/45690-12/2019(H3N1, G12) | H3_2000.3 | 89% |  |  |  | G | F | N |  |
| A/wild boar/Spain/45560-1/2022(H3N1, G12) | H3_2000.3 | 88% |  |  |  | G | F | N |  |

**Tabla S5. Hemagglutinin (HA) 3 antigenic variation.** Amino acid substitutions in HA3 of contemporary swine influenza strains are shown in comparison to the human seasonal H3N2 vaccine strain (HuVacH3). G: influenza A virus genotypes previously described in Encinas et al., 2022, PMID: 35039784. Percent identity refers to the full-length HA protein. Substitutions are indicated at antigenic sites within the receptor binding domain (RBD), specifically at H3-numbered positions: 145, 155, 156, 158, 159, 189, and 193. **^†^** Antigenic site numbering based on the complete HA sequence from the first methionine. Conservative substitutions are shaded dark grey; non-conservative substitutions are shaded light grey. White boxes correspond to identical residues.
